# Influence of childhood trauma and post-traumatic stress symptoms on impulsivity: focusing on differences according to the dimensions of impulsivity

**DOI:** 10.1080/20008198.2020.1796276

**Published:** 2020-08-06

**Authors:** Ji Hye Kim, Ji Young Choi

**Affiliations:** a Department of Psychiatry, Sanggye Paik Hospital, Inje University, Seoul, South Korea; b Department of Child Studies, Inha University, Incheon, South Korea

**Keywords:** childhood trauma, post-traumatic stress disorder symptoms, impulsivity, UPPS-P, trauma infantil, síntomas de estrés postraumático, impulsividad, UPPS-P, Análisis SEM, 童年期创伤, 创伤后应激症状, 冲动性, UPPS-P, SEM分析, • Impulsivity is conceptualized as a multidimensional construct associated with a broad range of impulsive behaviors. • Childhood trauma predicted the impulsivity dimensions of positive and negative urgency, lack of premeditation, and perseverance. • Among the multidimensional impulsivity constructs, PTSD symptoms associated with childhood trauma were particularly related to urgency. • Impulsive behaviors that stem from urgency may be better served by addressing difficulties that are associated with childhood trauma and PTSD symptoms.

## Abstract

**Background:**

Impulsivity, a trait and multidimensional construct, is associated with a wide range of impulsive behaviours. Although it is well documented that childhood trauma (CT) affects impulsivity, few studies examine whether its effects depend on particular dimensions of impulsivity and the role post-traumatic stress symptoms play in the relationship between childhood trauma and different dimensions of impulsivity.

**Objective:**

This research aims to explore the relationships between CT, PTSD, and impulsivity in a heterogeneous clinical sample. We also sought to examine whether the influence of CT on impulsivity differs across the dimensions of impulsivity.

**Method:**

We investigated the relationships between CT, symptoms of post-traumatic stress disorder (PTSD), and five dimensions of impulsivity using a sample of 162 non-psychotic psychiatric patients without neurocognitive diagnoses. Participants completed the Childhood Trauma Questionnaire (CTQ), Impact of Event Scale – Revised (IES), and the UPPS-P Impulsive Behaviour Scale (UPPS-P).

**Results:**

The results of structural equation modelling showed that CT is associated with PTSD symptoms, in addition to four of the five dimensions of impulsivity in the UPPS-P:positive urgency, negative urgency, lack of premeditation, and lack of perseverance. The indirect effect of CT through PTSD symptoms was significant only for the two types of urgency.

**Conclusions:**

The results of this study suggest that interventions that aim to alleviate impulsive behaviour derived from high urgency should pay particular attention to the presence of CT and PTSD symptoms.

## Abstract (es)

**Antecedentes**: La impulsividad, como un rasgo, se asocia con una amplia gama de comportamientos impulsivos y es un constructo multidimensional. Aunque está bien documentado que el trauma infantil (TI) influye en la impulsividad, hay pocos estudios que han examinado si sus efectos dependen de una dimensión particular de la impulsividad y qué papel juegan los síntomas de estrés postraumático en la relación entre el trauma infantil y cada dimensión de la impulsividad.

**Método**: Investigamos las relaciones entre el TI, los síntomas del trastorno de estrés postraumático (TEPT) y las cinco dimensiones de la impulsividad utilizando una muestra de 162 pacientes psiquiátricos no psicóticos y sin diagnóstico neurocognitivo. Los participantes completaron el Cuestionario de trauma infantil (CTQ), la Escala de impacto del evento revisada (IES) y la Escala de comportamiento impulsivo UPPS-P (UPPS-P).

**Resultados**: Los resultados del modelo de ecuaciones estructurales mostraron que el TI conduce a síntomas de TEPT, además de las cuatro dimensiones de impulsividad de urgencia positiva y negativa, falta de premeditación y perseverancia. El efecto indirecto del TI a través de los síntomas del TEPT fue significativo solo para los dos tipos de urgencia.

**Conclusiones**: Los resultados de este estudio sugieren que las intervenciones que tienen como objetivo aliviar los comportamientos impulsivos derivados de una alta urgencia deben prestar especial atención a la presencia de TI y síntomas de TEPT.

## Abstract (zh)

**背景**: 冲动性作为一种特质, 与多种冲动行为有关, 是一个多维构念。尽管童年期创伤 (CT) 影响冲动性有据可查, 但很少有研究考查其影响是否取决于冲动性的特定维度, 以及创伤后应激症状在童年期创伤与冲动性各维度之间的关系中起什么作用。

**方法**: 我们使用一个162例未经神经认知诊断的非精神病性精神病患者的样本, 研究了CT, 创伤后应激障碍 (PTSD) 症状和冲动性五个维度之间的关系。参与者完成了童年期创伤问卷 (CTQ), 事件影响量表修订版 (IES) 和UPPS-P冲动行为量表 (UPPS-P) 。

**结果**: 结构方程模型结果表明, CT会导致PTSD症状, 以及正性和负性紧迫感, 缺少预先考虑和毅力的四个冲动性维度。CT通过PTSD症状的间接影响仅对两种类型的紧迫感显著。

**结论**: 本研究结果表明, 旨在减轻因高紧迫感引起的冲动行为的干预措施应特别注意CT和PTSD症状的存在。

## 1. Introduction

Impulsivity, a trait and multidimensional construct, is associated with a wide range of impulsive behaviours. High levels of impulsivity are a common characteristic and a significant predictor of problematic behaviours indicative of various mental disorders (Ehring, 2010; Schaefer et al., 2018; Williams & Hasking, 2010). In addition, previous neurocognitive studies have revealed that impulsivity is relatedto familial vulnerability to mental illness. Therefore, impulsivity has been considered to be a potential endophenotype for bipolar disorder (Lombardo et al., 2012), substance abuse (Ersche, Turton, Pradhan, Bullmore, & Robbins, 2010), and suicidal behaviour (Anestis, Bagge, Tull, & Joiner, 2011). In other words, impulsivity is one of the transdiagnostic factors underlying various psychopathologies.

The precise definition of impulsivity as a construct is difficult to delineate because of the heterogeneity of its component dimensions (Block, 1995; Fischer, Smith, & Cyders, 2008). Consequently, the construct of impulsivity has been inappropriately operationalized in related dimensions, and conversely, the operationalization of such dimensions has been incorrectly regarded as measures of impulsivity. To address such issues, Whiteside, Lynam, Miller, & Reynolds (2005) examined the multifaceted nature of impulsivity and proposed the following four-factor structure: urgency, (lack of) premeditation, (lack of) perseverance, and sensation seeking. Later, Smith et al. (2007) added a fifth factor, positive urgency, to the original model, and the resulting five-factor structure was named the UPPS-P model.

Previous studies have demonstrated that the UPPS-P dimensions are significant predictors of a broad range of psychiatric disorders characterized by impulsive behaviours. For example, lack of premeditation is associated with alcohol and drug use (Magid & Colder, 2007) and borderline personality disorder (Peters, Derefinko, & Lynam, 2017). Lack of perseverance, which is indicative of difficulties in maintaining attention for long periods, is strongly related to problematic drinking and aggressive behaviours (Dick et al., 2010; Latzman & Vaidya, 2013). Individuals who exhibit high levels of sensation seeking tendencies tend to show a high threshold for fear and low sensitivity to pain (Anestis et al., 2011; Netter, Hennig, & Roed, 1996). As a result, such individuals are vulnerable to pathological gambling (Estevez, Herrero-Fernández, Sarabia, & Jauregui, 2015), antisocial behaviour (Mann et al., 2017), and aggressive behaviours (Pérez Fuentes, Molero Jurado, Carrión Martínez, Mercader Rubio, & Gázquez, 2016). Positive and negative urgency are responses to emotional experiences, and both factors are strongly related to substance abuse (Smith & Cyders, 2016; Weiss, Tull, Sullivan, Dixon-Gordon, & Gratz, 2015), self-injurious behaviour without suicidal intent (Allen, Fox, Schatten, & Hooley, 2019), and borderline personality disorder (Bøen et al., 2014).

Previous studies have demonstrated a greater frequency of impulsive behaviours in individuals who report a history of childhood trauma (CT). For example, individuals with CT have been found to be at risk for a wide range of impulsive behaviours associated with borderline personality disorder (Bornovalova, Gratz, Delany-Brumsey, Paulson, & Lejuez, 2006; Figueroa & Silk, 1997), eating disorders (Corstorphine, Waller, Lawson, & Ganis, 2007), pathological gambling (Kausch, Rugle, & Rowland, 2006), self-harm behaviours (Arens, Gaher, & Simons, 2012), and substance abuse (Zlotnick, Donaldson, Spirito, & Pearlstein, 1997). Emotion dysregulation may underlie such an association between CT and impulsive behaviours (Dvir, Ford, Hill, & Frazier, 2014; Mandavia, Robinson, Bradley, Ressler, & Powers, 2016; Messman-Moore & Bhuptani, 2017). CT not only impairs the existing ability to control emotions, but it also prevents the overall development of emotion regulation over time (Schäfer et al., 2017; Swogger, You, Cashman-Brown, & Conner, 2011; Van Der Kolk, Roth, Pelcovitz, Sunday, & Spinazzola, 2005). The results of both prospective and retrospective studies have shown that emotion regulation plays an important role in impulsive behaviours of individuals who experience childhood trauma (Kim & Cicchetti, 2010; Weiss, Tull, Anestis, & Gratz, 2013). In this manner, CT may lead to the use of maladaptive strategies to cope with challenging emotions, thereby rendering individuals with CT vulnerable to impulsive behaviours.

Difficulties in emotion regulation are characteristic features of post-traumatic stress disorder (PTSD) symptomatology, which includes the suppression and avoidance of trauma-related stimuli (American Psychiatric Association, 2013). It can also trigger impulsive behaviours that may immediately alleviate negative affect in the short-term. Consequently, the association between negative emotions and impulsive behaviour is reinforced over time. High levels of arousal in individuals with PTSD may deplete their regulatory resources, thereby making it difficult for them to control other behaviours (Baumeister, 2003). A growing body of empirical literature suggests that individuals with PTSD show high levels of impulsivity. For example, patients with PTSD have been known to engage in various types of impulsive behaviours such as substance abuse (Brady, Back, & Coffey, 2004), antisocial behaviour (Weiss, Tull, Viana, Anestis, & Gratz, 2012), eating disorders (Holzer, Uppala, Wonderlich, Crosby, & Simonich, 2008), deliberate self-harm (Sacks, Flood, Dennis, Hertzberg, & Beckham, 2008), and risky sexual behaviour (Weiss, Walsh, DiLillo, Messman-Moore, & Gratz, 2019). Specifically, chronic PTSD symptoms can contribute to the development of emotion dysregulation, which can trigger severe and complex patterns of comorbidities (Messman-Moore & Bhuptani, 2017).

Previous studies on the relationship between CT and PTSD have found that chronic PTSD symptoms are associated with emotion dysregulation (Dvir et al., 2014). Furthermore, when individuals attempt to cope with their PTSD symptoms, they may fail to modulate arousaland alleviate negative emotions. This, in turn, may result in substance-related problems and self-harm behaviours (Weiss et al., 2013). Compared to the other dimensions of impulsivity, urgency and sensation seeking have been more strongly implicated in the mechanisms that underlie the relationship between impulsivity and PTSD symptoms (Price, Connor, & Allen, 2017). Furthermore, emotional abuse and negative urgency exacerbate PTSD symptoms, which in turn often lead to substance-related problems (Mirhashem et al., 2017). These results suggest that the experience of CT and the resulting chronic negative emotional states may influence the emotion-related dimensions of impulsivity, namely, positive and negative urgency.

Several studies have explored the relationship that impulsivity shares with CT and PTSD (Bøen et al., 2014; Contractor, Armour, Forbes, & Elhai, 2016). However, a few studies have simultaneously examined the relationship between CT, PTSD, and impulsivity (Mirhashem et al., 2017; Price et al., 2017), both of which only focused on the effect of urgency. Although there is a growing interest in delineating the particular dimension of impulsivity such as urgency that may underlie various psychopathologies, it is not clear whether the effects of CT are contingent on the other dimensions of impulsivity, or whether PTSD symptomatology mediates the relationships between CT and the different dimensions of impulsivity. In addition, previous studies have used samples that exhibit specific types of impulsive behaviours (e.g. substance abuse, addiction, or self-injury) rather than a clinical sample of individuals who are diagnosed with a wide range of psychiatric disorders (Moeller, Barratt, Dougherty, Schmitz, & Swann, 2001).

Considering that impulsivity is one of the transdiagnostic factors related to various psychopathologies, we investigated the relationships between CT, PTSD, and impulsivity in a heterogeneous clinical sample. We also sought to examine whether the influence of CT on impulsivity differs across the dimensions of impulsivity. In particular, we used structural equation modelling (SEM) to examine the mediating effect of PTSD symptoms on the relationships between CT and each dimension of impulsivity.

## 2. Method

### 2.1. Participants and procedure

The sample consisted of 198 patients who received psychiatrictreatment at the university-affiliated hospital in Seoul. The diagnostic exclusion criteria were as follows: a) individuals with a diagnosis of psychotic symptoms (e.g. schizophrenia spectrum disorders, mood disorders with psychotic symptoms); b) individuals with a diagnosis of major neurocognitive disorders; and c) individualsdiagnosed with intellectual disability. A total of 162 adults (69 men, 93 women) between the ages of 18 and 81 years (*M* = 40.93, *SD *= 17.66) were included in the final sample. The DSM-5 was used to determine diagnoses for each participant. Primary psychiatric diagnoses among the participantswere as follows: depressive disorders (*n* = 81), anxiety disorders (*n* = 56), and other mental disorders (*n* = 25), which included PTSD, disruptive behaviour, impulse control, conduct disorder, substance use disorder, obsessive-compulsive disorder, and personality disorder. In addition, 35.2% of the participants (*n* = 57) received a dual diagnosis. The total number of participants who had PTSD was 12 (nine had a primary diagnosis of PTSD, and three had a secondary diagnosis of PTSD). The most common comorbidity that accompanied PTSD was depressive disorder (*n* = 8), followed by anxiety disorder (*n* = 8), and alcohol use disorder (*n* = 1). Informed consent was obtained from allparticipants following procedures approved by the hospital’s InstitutionalReview Board.

### 2.2. Measures

#### 2.2.1. The Korean childhood trauma questionnaire (K-CTQ)

The K-CTQ is a 28-item retrospectiveself-report questionnaire that assesses five types of childhood maltreatment: physical abuse, sexual abuse, emotional abuse, physical neglect, and emotional neglect (Bernstein & Fink, 1997). The composite score ranges from 28 to 140, with higher scores indicating greater severity of childhood maltreatment. A Korean validation study reported the Cronbach’s alpha coefficients for the five subscalesas follows: physical abuse0.82), sexual abuse (0.79),emotional abuse (0.80), physical neglect (0.51), and emotional neglect (0.89) (Yu, Park, Park, & Ryu, 2009). In the present study, the internal consistency results for the five subscales of theK-CTQ were as follows: physical abuse (0.86), sexual abuse (0.82), emotional abuse (0.82), physical neglect (0.68), and emotional neglect (0.88). In this study, scores on the emotional abuse, physical abuse, and sexual abuse subscales were used as the indicators of CT in SEM because neglect has not been as consistently associated with PTSD as abuse.

#### 2.2.2. The Korean version of the impact of event scale-revised (IES-R-K)

The Impact of Event Scale (IES) was developed by Horowitz et al. (Horowitz, Wilner, & Alvarez, 1979) as a self-report measure to assessthe central features of PTSD (e.g. trauma-related symptoms of intrusion and avoidance). We used the revised version of the assessment by Weiss and Marmar (2007), which includes symptoms of hyperarousal (i.e. IES-R). The IES-R consists of 22 items measured with a five-point Likert scale that includes the following subscales: intrusion, avoidance, numbing and dissociation, and hyperarousal. Validation of a Korean version of the IES-R (IES-R-K) was examined by Eun, Kwon, & Lee (2005). They showed the internal consistencies of the subscales ranged from 0.69 to 0.83. The Cronbach’s alpha coefficients of the subscales in the present study are as follows: intrusion (0.92), avoidance (0.93), numbing and dissociation (0.83), and hyperarousal (0.91).

#### 2.2.3. The UPPS-P impulsive behaviour scale

To measure impulsivity facets, the UPPS-P Impulsive Behaviour Scale was used (Cyders et al., 2007; Whiteside et al., 2005). It is a 59-item inventory with a four-point Likert scale that assesses five dimensions of impulsivity: positive and negative urgency, lack of perseverance, lack of premeditation, and sensation seeking. The Korean version of this tool has demonstrated good reliability (α = 0.78–0.92) and external validity (Sun Young & Young Ho, 2014). In the present study, the internal consistency coefficients of the subscales ranged from 0.83 for negative urgency (very good reliability) to.91 for positive urgency (excellent reliability).

### 2.3. Statistical analyses

A partial correlation analysis was performed to examine the relationship between CT, PTSD symptoms, and multidimensional impulsivity. We used the maximum likelihood estimation of SEM modelsto examine the mediating effect of PTSD symptoms on the relationship between CT and impulsivity. The entire mediation model with a direct path from CT to each facet of impulsivity was assessed. The comparative fit index (CFI), Bentler-Bonett Normed Fit Index (NFI), and root mean square error of approximation (RMSEA) were usedto examine model fit. Conventionally, CFI and NFI values that are greater than 0.90 and an RMSEA value that is closer to zero are indicative of an acceptable model fit. To determine the significance of the mediating and indirect effects, the bias-corrected percentile confidence intervals that were yielded by 1,000 bootstrap iterationswere used (Preacher & Hayes, 2008). If a value of zero did not feature within the 95% confidence intervals of the bootstrap samples, the mediation effects were considered to be statistically significant. All analyses were conducted with AMOS 21.0 (Arbuckle, 2012).

## 3. Results

### 3.1. Partial correlation analysis

The results of the partial correlation analysis and descriptive statistics (i.e. means and standard deviations, skewness) are presented in Table 1. With age and gender controlled, the results showed that the three dimensions of CT, namely, emotional, physical, and sexual abuse, were significantly correlated with the PTSD symptoms hyperarousal, avoidance, intrusion, and numbing and dissociation (*r* = 0.16–0.29, *p* < 0.05). The UPPS-P dimensions showed different patterns of correlations with CT and PTSD symptoms: positive and negative urgency were significantly correlated with all the subscales of the assessments that were used to measure CT and PTSD (*r* = 0.33–0.45, *p* < 0.001). Lack of premeditation and lack of perseverance were not significantly related to PTSD symptoms, but they were related to CT. Sensation seeking was significantly related to PTSD symptoms but not CT.

**Table 1.** Partial correlations among the study variables.

Variable	PA	SA	EA	HA	AV	IN	ND	NU	PU	LPM	LPS	SS
PA	-	0.53**	0.83**	0.29**	0.21**	0.26**	0.25**	0.25**	0.22*	0.14	0.12	0.09
SA		-	0.57**	0.23**	0.22**	0.21**	0.16**	0.28**	0.27**	0.09	0.05	−0.08
EA			-	0.27**	0.19**	0.27**	0.25**	0.25***	0.18*	0.19*	0.19*	−0.04
HA				-	0.83**	0.92**	0.83**	0.40***	0.37***	0.06	0.05	0.38***
AV					-	0.84**	0.74**	0.38***	0.33***	−0.02	−0.01	0.41***
IN						-	0.80**	0.39***	0.34***	0.08	0.04	0.33***
ND							-	0.46***	0.42***	0.02	0.05	0.42***
NU								-	0.88***	0.33***	0.31***	0.38***
PU									-	0.22*	0.17	0.39***
LPM										-	0.71***	−0.07
LPS											-	−0.27**
SS												
*M*	15.56	13.68	10.66	8.06	6.05	7.90	11.72	39.48	51.12	38.55	33.45	46.34
*SD*	8.91	7.90	5.94	5.71	3.98	5.14	5.06	11.59	13.05	8.76	7.47	9.75
*Skewness* *(SE)*	0.92 (0.19)	1.45 (0.19)	0.74 (0.19)	−0.04 (0.19)	−0.09 (0.19)	−0.16 (0.19)	0.10 (0.19)	0.14 (0.21)	0.26 (0.21)	0.47 (0.21)	−0.25 (0.21)	0.45 (0.21)

PA = Physical Abuse; SA = Sexual Abuse; EA = Emotional Abuse; HA = Hyperarousal; AV = Avoidance; IN = Intrusion; ND = Numbing and Dissociation; NU = Negative Urgency; PU = Positive Urgency; LPM = Lack of Premeditation; LPS = Lack of Perseverance; SS = Sensation Seeking; *M* = Mean; *SD* = Standard Deviation; *SE* = Standard Error. *p <0.05; **p <0.01; ***p <0.001.

^a^Age and gender were controlling covariates.

### 3.2. SEM

To examine the mediating effect of PTSD symptoms on the relationship between CT and impulsivity, SEM was conducted. First, the full mediation model was tested. More specifically, we examined if PTSD symptoms fully mediated the relationship between CT and the five dimensions of impulsivity. Next, a partial mediation model, which entailed direct paths from CT to each dimension of impulsivity, was compared to the full mediation model (Kline, 2005). The coefficients for the direct path from CT to four dimensions of the UPPS-P (excluding sensation seeking) were significant in the partial mediation model. The results of both the full and partial mediation models demonstrated an acceptable fit: χ^2^(*df *= 43, *N* = 162) = 66.17, *p* < 0.05; CFI = 0.98; NFI = 0.96; RMSEA = 0.058 (90% CI = 0.027–0.084), χ^2^(*df *= 38, *N* = 162) = 52.70, *p* = 0.057; CFI = 0.99; NFI = 0.98; RMSEA = 0.049 (95% CI = 0.00–0.08). However, the chi-square difference test between the full and partial mediation model significantly decreased in fit: Δχ^2^ (5) = 13.47, *p* < 0.001. Thus, the partial mediation model was deemed more appropriate in examining the relationship between CT, PTSD symptoms, and the five dimensions of impulsivity.

CT was associated with PTSD (*β *= 0.43, *p* < 0.001), which in turn was related to negative urgency (*β *= 0.41, *p* < 0.001), positive urgency (*β *= 0.35, *p* < 0.001), and sensation seeking (*β *= 0.45, *p* < 0.001). CT was also directly related to negative urgency (*β *= 0.25, *p* < 0.01) and positive urgency (*β *= 0.18, *p* < 0.05). CT had a significant and direct effect on lack of premeditation (*β *= 0.25, *p* < 0.05) and lack of perseverance (*β *= 0.28, *p* < 0.01). However, the indirect path from PTSD symptoms to lack of premeditation and lack of perseverance was not significant. The coefficients for the direct paths from CT to sensation seeking also were not significant. However, the path from PTSD symptoms to sensation seeking was significant (*β *= 0.45, *p* < 0.001).

**Figure 1.**
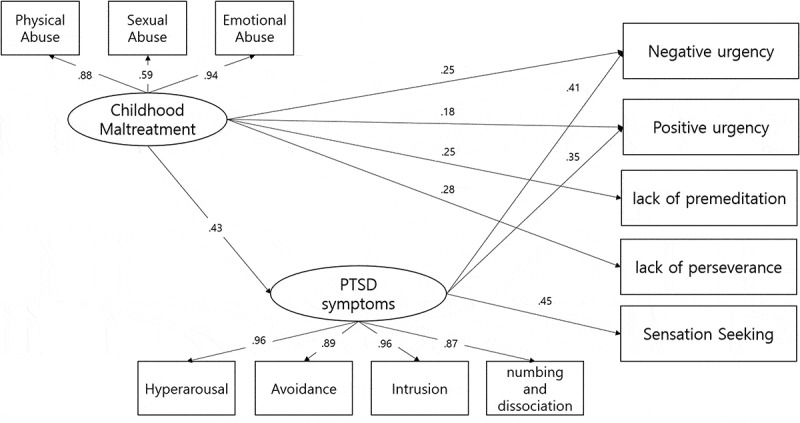
The partial mediation model (*N* = 162) and standardized path coefficients.

*Note*. PA = Physical Abuse; EA = Emotional Abuse; SA = Sexual Abuse; IES = Post traumatic Stress Symptoms; HA = Hyperarousal; AV = Avoidance; IN = Intrusion; ND = Numbing and Dissociation; LPM = Lack of Premeditation; SS = Sensation Seeking; PU = Positive Urgency; NU = Negative Urgency; LPS = Lack of Perseverance. **p <0.01; ***p <0.001.

Bootstrap 95% confidence intervals that do not include a zero are indicative of significant indirect effects. An examination of the indirect effect of CT on negative urgency through PTSD symptoms yielded the following results: *β *= 0.16, 95% CI = 0.08–0.25. Similarly, an examination of the indirect effect of CT on positive urgency through PTSD symptoms yielded the following results: *β *= 0.13, 95% CI = 0.06–0.24. These results are illustrated in Figure 1.

## 4. Discussion

Impulsivity is a multidimensional construct that underlies a number of psychiatric disorders characterized by a broad range of impulsive behaviours, and each dimension may play a unique role in the onset and maintenance of such psychiatric disorders (Berg, Latzman, Bliwise, & Lilienfeld, 2015; Derefinko, Dewall, Metze, Walsh, & Lynam, 2011; Johnson, Carver, & Tharp, 2017; Whiteside et al., 2005). On the other hand, environmental factors such as CT may exert differentialeffects on particular dimensions of impulsivity. Accordingly, we examined the different patterns of relationships that may exist between CT and the five dimensions of impulsivity. Furthermore, as previous studies have demonstrated that past CT and PTSD symptomsareassociated with facets of impulsivity (Contractor et al., 2016; Schaefer et al., 2018), we explored the role of PTSD symptoms in the relationship between CT and the different dimensions of impulsivity.

The results of the partial correlation analysis showed that four dimensions of impulsivity (excluding sensation seeking) were positively correlated with CT. However, their unique associations with CT and PTSD symptoms were different in the SEM models when the shared variance was accounted for. Specifically, CT predicted positive and negative urgency, lack of premeditation, and perseverance but was unrelated to sensation seeking. The mediating effect of PTSD symptoms was significant only for the association between CT and urgency. Furthermore, sensation seeking was related to PTSD symptoms but not to CT.

The present findings thus revealed unique patterns of relationships that exist between CT, PTSD symptoms, and the five dimensions of impulsivity. A recent examination of the factor structure of the UPPS-P demonstrated support for a hierarchical four-factor model instead of the five-factor model. In this model,lack of premeditation and lack of perseverance were loaded onto one higher-order factor labelled lack of conscientiousness. On the other hand, urgency and sensation seeking were independent, albeit correlated factors (Billieux, Lagrange, & der L-P, 2012; Smith et al., 2007). Although we did not examine the hierarchical model of impulsivity, it is clear that there were three distinct patterns of impulsivity in the relationship between CT and PTSD.

The indirect effect of CT through PTSD symptoms was significant only for the two types of urgency. These findings are consistent with studies on the mechanism underlying impulsive behaviours with a specific focus on the role of emotion dysregulation in PTSD. According to the theoretical literature on emotion dysregulation, traumatic experiences exacerbate physiological responses to negative emotions and trauma-related cues. This leads to impulsivity and poor perseverance (Weiss et al., 2013). Specifically, when compared to normal controls, patients with PTSD exhibit a higher number of impulsive behaviours (e.g. smoking, self-harm) when they experience and process heightened arousal and negative emotions. This behavioural pattern is also significantly correlated with negative urgency and sensation seeking (Contractor et al., 2016; Marshall-Berenz, Vujanovic, & MacPherson, 2011). Furthermore, urgency, sensation seeking, and emotion dysregulation are significantly related to PTSD symptoms (Weiss et al., 2015). The findings of the present study demonstrate that CTmay account for PTSD symptoms, which in turn may particularly increase impulsivity in the domains of urgency. These findings suggest that PTSD symptoms play a role in emotion-related impulsivity, especially in overall self-control. A similar pattern of relationship that the two strongly related factors ofnegative and positive urgency share with CT and PTSD suggests that these two dimensions of impulsivity may be conceptualized as a unitary construct. Smith et al. (2007) conducted a confirmatory factor analysis on the UPPS-P and found that negative and positive urgency are facets of a common trait wherein both are related to emotionality and affective dysregulation. Moreover, each of the two types of urgency distinctively predicted risky behaviours.

Our results concur with previous findings that urgency, rather than lack of premeditation and perseverance, is the dimension of impulsivity that may be most relevant to PTSD symptoms (Roley, Contractor, Weiss, Armour, & Elhai, 2017). A meta-analytic review that compared the UPPS-P dimensions and a range of psychiatric disorders (Berg et al., 2015) showed that, unlike the other dimensions of impulsivity, lack of perseverance and premeditation did not have a significant effect on anxiety disorders. A recent study also supported this pattern of relationships between PTSD symptoms and the UPPS-P dimensions. Both negative urgency and sensation seeking were significantly related to PTSD symptoms but lack of perseverance and premeditation were unrelated to PTSD (Contractor et al., 2016). It is possible that variables other than PTSD symptoms (e.g. executive function) affected the relationship between CT and lack of premeditation and perseverance. These two dimensions are conceptually similar to the construct of conscientiousness, a personality trait that is characterized by self-discipline (Whiteside et al., 2005). These dimensions of impulsivity may impair one’s ability to foresee long-term consequences of their actions. Dawe and Loxton (2004) also referred to an association between decision-making and unplanned impulsivity rather than reward sensitivity or drive. Some empirical findings have supported the link between these two dimensions of impulsivity and executive function. For example, lack of premeditation significantly predicted poor decision-making during the Iowa Gambling Task (Zermatten, Van Der Linden, D’Acremont, Jermann, & Bechara, 2005). In another study, working memory moderated the association between personality traits related to impulsivity and alcohol problems (Ellingson, Fleming, Vergés, Bartholow, & Sher, 2014; Finn & Hall, 2004). Since childhood maltreatment can impair executive function (Hart & Rubia, 2012; Mothes et al., 2015; Spann et al., 2012), it is possible that executive function mediates the association between CT and lack of premeditation and lack of perseverance. In particular, severe CT during the early stages of development interferes with the maturation of the frontal lobe, which results in impulsive behaviours such as drinking and drug abuse during adolescence (Van Harmelen et al., 2010). As executive functions are involved in emotion regulation and decision-making behaviour, it may be necessary to further explore the influence of psychobiological factors on the relationship between lack of premeditation, lack of perseverance, and CT.

In contrast to lack of premeditation and perseverance, which were both associated only with CT and not PTSD, sensation seeking was associated with PTSD symptoms but not with CT. Previous studies have consistently found a relationship between sensation seeking and PTSD symptoms. For example, Roley et al. (2017) found that sensation seeking was positively correlated with all the symptoms of PTSD as specified in the DSM-5 except for intrusive symptoms. In addition, Contractor et al. (2016) reported that out of all the symptoms of PTSD, negative alterations in mood and cognition and alterations in arousal and reactivity were most strongly correlated with sensation seeking. However, in the present study, the correlation between sensation seeking and CT was not significant. Bøen et al. (2014) compared the profiles of impulsivity of individuals who had been diagnosed with borderline personality disorder and bipolar disorder. The results showed that sensation seeking was unrelated to CT in both groups. Our results provide a possible explanation for why sensation seeking was correlated with PTSD symptoms but not with CT because we simultaneously analysed all three variables: CT, PTSD, and impulsivity. Sensation seeking is a predictor of delinquent behaviours during adolescence and antisocial personality disorder. Also, sex and age differences were more pronounced for sensation seeking than for the other dimensions of impulsivity (Dir, Coskunpinar, & Cyders, 2014). Thus, when compared to the other dimensions of impulsivity, sensation seeking may be affected to a greater extent by genetic rather than environmental factors (Mann et al., 2017). Those who are genetically predisposed to engage in sensation seeking behaviours may be more vulnerable to externalizing behaviours that alleviate negative emotions. Another possible explanation is that sensation seeking may be related to other kinds of traumatic experiences that are not measured in this study. Other types of traumatic events experienced in childhood, not maltreatment or life-threatening experiences with physical pain, regardless of the time of experience, may be associated with sensationseeking. Further research would be needed to explore the relationship between sensation seeking and a broader range of traumatic experiences.

The present study has a few limitations. First, we attempted to examine the association between CT, PTSD, and impulsivity using a heterogeneous sample of psychiatric patients, but a majority of the participants were diagnosed with depressive and anxiety disorders and a relatively smaller portion of the sample was diagnosed with PTSD. Therefore, the findings of the present study may be difficult to generalize to individuals with a primary diagnosis of PTSD or other diagnoses that are characterized by impulsive behaviours. In order to address this issue, future studies should recruit a higher number of participants with PTSD and impulsivity-related disorders to replicate the results of this study. Second, we did not examine any other potential mediating variables other than PTSD symptoms. For example, executive function may affect the relationship between CT and lack of premeditation and perseverance. Given the critical role of urgency in CT and PTSD, it may also be important to examine the extent to which this relationship is distinct from high levels of neuroticism. For instance, a recent study reported that urgency may account for the variance in impulsive behaviour that is not accounted for by neuroticism (Settles et al., 2012), despite the shared variance between urgency and neuroticism. Both of the constructs may be a part of emotion-related impulsivity, but they may play a different role in the relationship between CT and impulsive behaviours. Third, the cross-sectional nature of the present study does not allow us to draw inferences regarding the causality of the mediation effects that were observed. Previous longitudinal studies have found that early and repeated exposure to CT leads to a heightened level of urgency, which increases the likelihood of engaging in impulsive behaviours such as drinking and substance abuse (Mirhashem et al., 2017). Therefore, the relationship between CT, PTSD, and impulsivityneeds to be examined using longitudinal research designs. Finally, impulsivity was measured using a self-report measure which is vulnerable to response bias. Therefore, future studies should employ alternative strategies to measure impulsivity. Specifically, the inclusion ofbehavioral data as well as theoretical concepts to measure the frequency and intensity of impulsive behaviorsis needed.

## 5. Conclusion

Despite such limitations, the present study makes noteworthy contributions to the literature. Specifically, the findings suggest that the relationship between CT, PTSD symptoms, and impulsivity are contingent on the specific dimension of impulsivity. In particular, this study showed that PTSD symptoms may play a significant role inthe relationship between CT and urgency.

Our findings have several implications for clinicians working with impulsive individuals. They suggest that clinicians not only need to understand the specific dimensions of impulsivity leading to impulsive behaviours but also should pay attention to the individual’s history that may be related to the identified dimensions. In particular, it would be useful to include a process for exploring and dealing with childhood trauma and PTSD symptoms in treatment plans for individuals with high urgency. Addressing PTSD symptoms can also help alleviate one’s impulsive behaviours resulting from urgency and sensation seeking. This approach to patients with a high level of impulsivity could be a potential option for the treatment of impulsive behaviour.

## References

[cit0001] Allen, K. J. D., Fox, K. R., Schatten, H. T., & Hooley, J. M. (2019). Frequency of nonsuicidal self-injury is associated with impulsive decision-making during criticism. *Psychiatry Research*, 271, 68–10. 30469091 10.1016/j.psychres.2018.11.022 PMC6382530

[cit0002] American Psychiatric Association. (2013). DSM-5 diagnostic classification. *Diagnostic and Statistical Manual of Mental Disorders*, doi:10.1176/appi.books.9780890425596.x00diagnosticclassification.

[cit0003] Anestis, M. D., Bagge, C. L., Tull, M. T., & Joiner, T. E. (2011). Clarifying the role of emotion dysregulation in the interpersonal-psychological theory of suicidal behavior in an undergraduate sample. *Journal of Psychiatric Research*, 45(5), 603–611. 21092986 10.1016/j.jpsychires.2010.10.013

[cit0004] Arbuckle, J. L. (2012). *IBM SPSS Amos 21 user ’ s guide*. Crawfordville, FL: Amos Dev. doi:10.1111/j.1600-0447.2011.01711.x

[cit0005] Arens, A. M., Gaher, R. M., & Simons, J. S. (2012). Child maltreatment and deliberate self-harm among college students: Testing mediation and moderation models for impulsivity. *American Journal of Orthopsychiatry*, 82(3), 328–337. 10.1111/j.1939-0025.2012.01165.x 22880971

[cit0006] Baumeister, R. F. (2003). Ego depletion and self-regulation failure: A resource model of self-control. *Alcoholism: Clinical & Experimental Research*, 27(2), 281–284. 10.1097/01.ALC.0000060879.61384.A4 12605077

[cit0007] Berg, J. M., Latzman, R. D., Bliwise, N. G., & Lilienfeld, S. O. (2015). Parsing the heterogeneity of impulsivity: A meta-analytic review of the behavioral implications of the UPPS for psychopathology. *Psychological Assessment*, 27(4), 1129–1146. 25822833 10.1037/pas0000111

[cit0008] Bernstein, D. P., & Fink, L. (1997). Childhood trauma questionnaire: A retrospective self-report (CTQ). *Pearson*. doi:10.1126/science.233.4768.1102

[cit0009] Billieux, J., Lagrange, G., & der L-P, M. V. (2012). Investigation of impulsivity in a sample of treatment-seeking pathological gamblers: A multidimensional perspective. *Psychiatry Research*, 198(2), 291–296. 10.1016/j.psychres.2012.01.001 22421073

[cit0010] Block, J. (1995). A contrarian view of the five-factor approach to personality description. *Psychological Bulletin*, 117(2), 187. 7724687 10.1037/0033-2909.117.2.187

[cit0011] Bøen, E., Hummelen, B., Elvsåshagen, T., Boye, B., Andersson, S., Karterud, S., & Malt, U. F. (2014). Different impulsivity profiles in borderline personality disorder and bipolar II disorder. *Journal of Affective Disorders*, 170C, 104–111. 10.1016/j.jad.2014.08.033 25237733

[cit0012] Bornovalova, M. A., Gratz, K. L., Delany-Brumsey, A., Paulson, A., & Lejuez, C. W. (2006). Temperamental and environmental risk factors for borderline personality disorder among inner-city substance users in residential treatment. *Journal of Personality Disorders*, 20(3), 218–231. 16776552 10.1521/pedi.2006.20.3.218

[cit0013] Brady, K. T., Back, S. E., & Coffey, S. F. (2004). Substance abuse and posttraumatic stress disorder. *Current Directions in Psychological Science*, 13(5), 206–209.

[cit0014] Contractor, A. A., Armour, C., Forbes, D., & Elhai, J. D. (2016). Posttraumatic stress disorder’s underlying dimensions and their relation with impulsivity facets. *The Journal of Nervous and Mental Disease*, 204(1), 20–25. 26558499 10.1097/NMD.0000000000000417

[cit0015] Corstorphine, E., Waller, G., Lawson, R., & Ganis, C. (2007). Trauma and multi-impulsivity in the eating disorders. *Eating Behaviors*, 8(1), 23–30. 17174848 10.1016/j.eatbeh.2004.08.009

[cit0016] Cyders, M., Fischer, S., Peterson, C. M., Cyders, M. A., Smith, G. T., Fischer, S., Annus, A. M., & Peterson, C. 2007. Integration of impulsivity and positive mood to predict risky behavior : Development and validation of a measure of positive urgency. *PsycnetApaOrg*. doi:10.1037/1040-3590.19.1.107 17371126

[cit0017] Dawe, S., & Loxton, N. J. (2004). The role of impulsivity in the development of substance use and eating disorders. *Neuroscience & Biobehavioral Reviews*, 28(3), 343–351. 15225976 10.1016/j.neubiorev.2004.03.007

[cit0018] Derefinko, K., Dewall, C. N., Metze, A. V., Walsh, E. C., & Lynam, D. R. (2011). Do different facets of impulsivity predict different types of aggression? *Aggressive Behavior*, 37(3), 223–233. 21259270 10.1002/ab.20387 PMC3063858

[cit0019] Dick, D. M., Smith, G., Olausson, P., Mitchell, S. H., Leeman, R. F., O’Malley, S. S., & Sher, K. (2010). Understanding the construct of impulsivity and its relationship to alcohol use disorders. *Addiction Biology*, 15(2), 217–226. 20148781 10.1111/j.1369-1600.2009.00190.x PMC2895996

[cit0020] Dir, A. L., Coskunpinar, A., & Cyders, M. A. (2014). A meta-analytic review of the relationship between adolescent risky sexual behavior and impulsivity across gender, age, and race. *Clinical Psychology Review*, 34(7), 551–562. 25261740 10.1016/j.cpr.2014.08.004

[cit0021] Dvir, Y., Ford, J. D., Hill, M., & Frazier, J. A. (2014). Childhood maltreatment, emotional dysregulation, and psychiatric comorbidities. *Harvard Review of Psychiatry*, 22(3), 149–161. 24704784 10.1097/HRP.0000000000000014 PMC4091823

[cit0022] Ehring, T., & Quack, D. (2010). Emotion regulation difficulties in trauma survivors: The role of trauma type and PTSD symptom severity. *Behavior Therapy*, 41(4), 587–598. 21035621 10.1016/j.beth.2010.04.004

[cit0023] Ellingson, J. M., Fleming, K. A., Vergés, A., Bartholow, B. D., & Sher, K. J. (2014). Working memory as a moderator of impulsivity and alcohol involvement: Testing the cognitive-motivational theory of alcohol use with prospective and working memory updating data. *Addictive Behaviors*, 39(11), 1622–1631. 24508184 10.1016/j.addbeh.2014.01.004 PMC4108580

[cit0024] Ersche, K. D., Turton, A. J., Pradhan, S., Bullmore, E. T., & Robbins, T. W. (2010). Drug addiction endophenotypes: Impulsive versus sensation-seeking personality traits. *Biological Psychiatry*, 68(8), 770–773. 20678754 10.1016/j.biopsych.2010.06.015 PMC3485555

[cit0025] Estevez, A., Herrero-Fernández, D., Sarabia, I., & Jauregui, P. (2015). The impulsivity and sensation-seeking mediators of the psychological consequences of pathological gambling in adolescence. *Journal of Gambling Studies*, 31(1), 91–103. 24297606 10.1007/s10899-013-9419-0

[cit0026] Eun, H., Kwon, T., & Lee, S. (2005). A study on reliability and validity of the Korean version of impact of event scale-revised. *JKNA*, 44, 303–310.

[cit0027] Figueroa, E., & Silk, K. R. (1997). Biological implications of childhood sexual abuse in borderline personality disorder. *Journal of Personality Disorders*, 11(1), 71–92. 9113823 10.1521/pedi.1997.11.1.71

[cit0028] Finn, P. R., & Hall, J. (2004). Cognitive ability and risk for alcoholism: Short-term memory capacity and intelligence moderate personality risk for alcohol problems. *Journal of Abnormal Psychology*, 113(4), 569–581. 15535789 10.1037/0021-843X.113.4.569

[cit0029] Fischer, S., Smith, G. T., & Cyders, M. A. (2008). Another look at impulsivity: A meta-analytic review comparing specific dispositions to rash action in their relationship to bulimic symptoms. *Clinical Psychology Review*, 28(8), 1413–1425. 18848741 10.1016/j.cpr.2008.09.001 PMC2677964

[cit0030] Hart, H., & Rubia, K. (2012). Neuroimaging of child abuse: A critical review. *Frontiers in Human Neuroscience*, 6. doi:10.3389/fnhum.2012.00052 22457645 PMC3307045

[cit0031] Holzer, S. R., Uppala, S., Wonderlich, S. A., Crosby, R. D., & Simonich, H. (2008). Mediational significance of PTSD in the relationship of sexual trauma and eating disorders. *Child Abuse & Neglect*, 32(5), 561–6. 18511117 10.1016/j.chiabu.2007.07.011

[cit0032] Horowitz, M., Wilner, N., & Alvarez, W. (1979). Impact of event scale: A measure of subjective stress. *Psychosomatic Medicine*, 41(3), 209–218. 472086 10.1097/00006842-197905000-00004

[cit0033] Johnson, S. L., Carver, C. S., & Tharp, J. A. (2017). Suicidality in bipolar disorder: The role of emotion-triggered impulsivity. *Suicide and Life-Threatening Behavior*, 47(2), 177–192. 27406282 10.1111/sltb.12274 PMC5788807

[cit0034] Kausch, O., Rugle, L., & Rowland, D. Y. (2006). Lifetime histories of trauma among pathological gamblers. *American Journal on Addictions*, 15(1), 35–43. 10.1080/10550490500419045 16449091

[cit0035] Kim, J., & Cicchetti, D. (2010). Longitudinal pathways linking child maltreatment, emotion regulation, peer relations, and psychopathology. *Journal of Child Psychology and Psychiatry*, 511(6), 706–16. 10.1111/j.1469-7610.2009.02202.x PMC3397665 20050965

[cit0036] Kline, T. (2005). *Psychological testing: A practical approach to design and evaluation*. London: SAGE. doi:10.4135/9781483385693

[cit0037] Latzman, R. D., & Vaidya, J. G. (2013). Common and distinct associations between aggression and alcohol problems with trait disinhibition. *Journal of Psychopathology and Behavioral Assessment*, 35(2), 186–196.

[cit0038] Lombardo, L. E., Bearden, C. E., Barrett, J., Brumbaugh, M. S., Pittman, B., Frangou, S., & Glahn, D. C. (2012). Trait impulsivity as an endophenotype for bipolar I disorder. *Bipolar Disorders*, 14(5), 565–570. 22805501 10.1111/j.1399-5618.2012.01035.x PMC3653436

[cit0039] Magid, V., & Colder, C. (2007). The UPPS impulsive behavior scale: Factor structure and associations with college drinking. *Personality and Individual Differences*, 43(7), 1927–1937.

[cit0040] Mandavia, A., Robinson, G. G. N., Bradley, B., Ressler, K. J., & Powers, A. (2016). Exposure to childhood abuse and later substance use: Indirect effects of emotion dysregulation and exposure to trauma. *Journal of Traumatic Stress*, 29(5), 422–429. 27622844 10.1002/jts.22131 PMC5064859

[cit0041] Mann, F. D., Engelhardt, L., Briley, D. A., Grotzinger, A. D., Patterson, M. W., Tackett, J. L., & Harden, K. P. (2017). Sensation seeking and impulsive traits as personality endophenotypes for antisocial behavior: Evidence from two independent samples. *Personality and Individual Differences*, 105, 30–39. 28824215 10.1016/j.paid.2016.09.018 PMC5560504

[cit0042] Marshall-Berenz, E. C., Vujanovic, A. A., & MacPherson, L. (2011). Impulsivity and alcohol use coping motives in a trauma-exposed sample: The mediating role of distress tolerance. *Personality and Individual Differences*, 50(5), 588–592. 21483650 10.1016/j.paid.2010.11.033 PMC3072811

[cit0043] Messman-Moore, T. L., & Bhuptani, P. H. (2017). A review of the long-term impact of child maltreatment on posttraumatic stress disorder and its comorbidities: An emotion dysregulation perspective. *Clinical Psychology: Science and Practice*, 24(2), 154–169.

[cit0044] Mirhashem, R., Allen, H. C., Adams, Z. W., van Stolk-cooke, K., Legrand, A., & Price, M. (2015). The intervening role of urgency on the association between childhood maltreatment, PTSD, and substance-related problems. *Addictive Behaviors*, 69, 98–103. 10.1016/j.addbeh.2017.02.012 PMC5384831 28219827

[cit0045] Mirhashem, R., Allen, H. C., Adams, Z. W., van Stolk-cooke, K., Legrand, A., & Price, M. (2017). The intervening role of urgency on the association between childhood maltreatment, PTSD, and substance-related problems. *Addictive Behaviors*, 69, 98–103. 28219827 10.1016/j.addbeh.2017.02.012 PMC5384831

[cit0046] Moeller, F. G., Barratt, E. S., Dougherty, D. M., Schmitz, J. M., & Swann, A. C. (2001). Psychiatric aspects of impulsivity. *American Journal of Psychiatry*, 158(11), 1783–93. 10.1176/appi.ajp.158.11.1783 11691682

[cit0047] Mothes, L., Kristensen, C. H., Grassi-Oliveira, R., Fonseca, R. P., de Lima Argimon, I. I., & Irigaray, T. Q. (2015). Childhood maltreatment and executive functions in adolescents. *Child and Adolescent Mental Health*, 20(1), 56–62. 32680329 10.1111/camh.12068

[cit0048] Netter, P., Hennig, J., & Roed, I. S. (1996). Serotonin and dopamine as mediators of sensation seeking behavior. *Neuropsychobiology*, 34(3), 155–165. 8916073 10.1159/000119318

[cit0049] Pérez Fuentes, M. D. C., Molero Jurado, M. D. M., Carrión Martínez, J. J., Mercader Rubio, I., & Gázquez, J. J. (2016). Sensation-seeking and impulsivity as predictors of reactive and proactive aggression in adolescents. *Frontiers in Psychology*, 7. 10.3389/fpsyg.2016.01447 27729883 PMC5037130

[cit0050] Peters, J. R., Derefinko, K. J., & Lynam, D. R. (2017). Negative urgency accounts for the association between borderline personality features and intimate partner violence in young men. *Journal of Personality Disorders*, 31(1), 16–25. 26845532 10.1521/pedi_2016_30_234 PMC4974142

[cit0051] Preacher, K. J., & Hayes, A. F. (2008). Asymptotic and resampling strategies for assessing and comparing indirect effects in multiple mediator models. *Behavior Research Methods*, 40(3), 879–891. 18697684 10.3758/brm.40.3.879

[cit0052] Price, M., Connor, J. P., & Allen, H. C. (2017). The moderating effect of childhood maltreatment on the relations among PTSD symptoms, positive urgency, and negative urgency. *Journal of Traumatic Stress*, 30(4), 432–437. 28700808 10.1002/jts.22198 PMC5874149

[cit0053] Roley, M. E., Contractor, A. A., Weiss, N. H., Armour, C., & Elhai, J. D. (2017). Impulsivity facets’ predictive relations with DSM-5 PTSD symptom clusters. *Psychological Trauma: Theory, Research, Practice, and Policy*, 9(1), 76–79. 10.1037/tra0000146 PMC5317014 27243571

[cit0054] Sacks, M. B., Flood, A. M., Dennis, M. F., Hertzberg, M. A., & Beckham, J. C. (2008). Self-mutilative behaviors in male veterans with posttraumatic stress disorder. *Journal of Psychiatric Research*, 42(6), 487–494. 17606271 10.1016/j.jpsychires.2007.05.001 PMC2441874

[cit0055] Schaefer, J. D., Moffitt, T. E., Arseneault, L., Danese, A., Fisher, H. L., Houts, R., … Caspi, A. (2018). Adolescent victimization and early-adult psychopathology: Approaching causal inference using a longitudinal twin study to rule out noncausal explanations. *Clinical Psychological Science*, 6(3), 352–371. 29805917 10.1177/2167702617741381 PMC5952301

[cit0056] Schäfer, I., Pawils, S., Driessen, M., Härter, M., Hillemacher, T., Klein, M., … & Schneider, B. (2017). Understanding the role of childhood abuse and neglect as a cause and consequence of substance abuse: The German CANSAS network. *European Journal of Psychotraumatology*, 8. doi:10.1080/20008198.2017.1304114 PMC5399994 28451071

[cit0057] Settles, R. E., Fischer, S., Cyders, M. A., Combs, J. L., Gunn, R. L., & Smith, G. T. (2012). Negative urgency: A personality predictor of externalizing behavior characterized by neuroticism, low conscientiousness, and disagreeableness. *Journal of Abnormal Psychology*, 121(1), 160–172. 21859164 10.1037/a0024948 PMC3299541

[cit0058] Smith, G. T., & Cyders, M. A. (2016). Integrating affect and impulsivity: The role of positive and negative urgency in substance use risk. *Drug and Alcohol Dependence*, 163, S3–12. 27306729 10.1016/j.drugalcdep.2015.08.038 PMC4911536

[cit0059] Smith, G. T., Fischer, S., Cyders, M. A., Annus, A. M., Spillane, N. S., & McCarthy, D. M. (2007). On the validity and utility of discriminating among impulsivity-like traits. *Assessment*, 14(2), 155–170. 17504888 10.1177/1073191106295527

[cit0060] Spann, M. N., Mayes, L. C., Kalmar, J. H., Guiney, J., Womer, F. Y., Pittman, B., & Blumberg, H. P. (2012). Childhood abuse and neglect and cognitive flexibility in adolescents. *Child Neuropsychology*, 18(2), 182–189. 21942637 10.1080/09297049.2011.595400 PMC3326262

[cit0061] Sun Young, L., & Young Ho, L. A. (2014). Korean validation of the UPPS-P impulsive behavior scale in college students. *Journal of Clinical Psychology*, 33, 51–71.

[cit0062] Swogger, M. T., You, S., Cashman-Brown, S., & Conner, K. R. (2011). Childhood physical abuse, aggression, and suicide attempts among criminal offenders. *Psychiatry Research*, 185(3), 363–367. 20724000 10.1016/j.psychres.2010.07.036 PMC3032000

[cit0063] Van Der Kolk, B. A., Roth, S., Pelcovitz, D., Sunday, S., & Spinazzola, J. (2005). Disorders of extreme stress: The empirical foundation of a complex adaptation to trauma. *Journal of Traumatic Stress*, 18(5), 389–99. 16281237 10.1002/jts.20047

[cit0064] Van Harmelen, A.-L., Van Tol, M.-J., Van Der Wee, N. J. A., Veltman, D. J., Aleman, A., Spinhoven, P., & Elzinga, B. M. (2010). Reduced medial prefrontal cortex volume in adults reporting childhood emotional maltreatment. *Biological Psychiatry*, 68(9), 832–838. 20692648 10.1016/j.biopsych.2010.06.011

[cit0065] Weiss, D. S., & Marmar, C. R. (2007). *The impact of event scale: Revised. Cross-cultural assess. Psychological Trauma PTSD* (pp. 219–238). Boston, MA: Springer US. doi:10.1007/978-0-387-70990-1_10

[cit0066] Weiss, N. H., Tull, M. T., Anestis, M. D., & Gratz, K. L. (2013). The relative and unique contributions of emotion dysregulation and impulsivity to posttraumatic stress disorder among substance dependent inpatients. *Drug and Alcohol Dependence*, 128(1–2), 45–51. 22917752 10.1016/j.drugalcdep.2012.07.017 PMC3513512

[cit0067] Weiss, N. H., Tull, M. T., Sullivan, T. P., Dixon-Gordon, K. L., & Gratz, K. L. (2015). Posttraumatic stress disorder symptoms and risky behaviors among trauma-exposed inpatients with substance dependence: The influence of negative and positive urgency. *Drug and Alcohol Dependence*, 155, 147–153. 26278196 10.1016/j.drugalcdep.2015.07.679 PMC4581985

[cit0068] Weiss, N. H., Tull, M. T., Viana, A. G., Anestis, M. D., & Gratz, K. L. (2012). Impulsive behaviors as an emotion regulation strategy: Examining associations between PTSD, emotion dysregulation, and impulsive behaviors among substance dependent inpatients. *Journal of Anxiety Disorders*, 26(3), 453–458. 22366447 10.1016/j.janxdis.2012.01.007 PMC3305816

[cit0069] Weiss, N. H., Walsh, K., DiLillo, D. D., Messman-Moore, T. L., & Gratz, K. L. (2019). A longitudinal examination of posttraumatic stress disorder symptoms and risky sexual behavior: Evaluating emotion dysregulation dimensions as mediators. *Archives of Sexual Behavior*, 48(3), 975–986. 30771054 10.1007/s10508-019-1392-y PMC6474756

[cit0070] Whiteside, S. P., Lynam, D. R., Miller, J. D., & Reynolds, S. K. (2005). Validation of the UPPS impulsive behaviour scale: A four-factor model of impulsivity. *European Journal of Personality*, 19(7), 559–574.

[cit0071] Williams, F., & Hasking, P. (2010). Emotion regulation, coping and alcohol use as moderators in the relationship between non-suicidal self-injury and psychological distress. *Prevention Science*, 11(1), 33–41. 19707873 10.1007/s11121-009-0147-8

[cit0072] Yu, J. H., Park, J. S., Park, D. H., & Ryu, S. H. H. J. (2009). Validation of the Korean childhood trauma questionnaire: The practical use in counselling and therapeutic intervention. *Korean Journal of Health Psychology*, 14(3), 563–578.

[cit0073] Zermatten, A., Van Der Linden, M., D’Acremont, M., Jermann, F., & Bechara, A. (2005). Impulsivity and decision making. *The Journal of Nervous and Mental Disease*, 193(10), 647–650. 16208159 10.1097/01.nmd.0000180777.41295.65

[cit0074] Zlotnick, C., Donaldson, D., Spirito, A., & Pearlstein, T. (1997). Affect regulation and suicide attempts in adolescent inpatients. *Journal of the American Academy of Child & Adolescent Psychiatry*, 36(6), 793–798. 9183134 10.1097/00004583-199706000-00016

